# Dietary Patterns, Type 2 Diabetes Awareness, and Anthropometric Risk Factors Among African American Students at a Historically Black College and University: A Cross-Sectional Study

**DOI:** 10.21203/rs.3.rs-10251974/v1

**Published:** 2026-08-11

**Authors:** Maryam Ganjavi, Joycelyn Peterson

**Affiliations:** Morgan State University

**Keywords:** type 2 diabetes, African American, HBCU, college students, nutrition literacy, dietary patterns, anthropometry, cross-sectional study

## Abstract

**Background:**

Type 2 diabetes (T2D) disproportionately affects African Americans, yet data on diabetes awareness, dietary behaviors, and anthropometric risk factors among students attending Historically Black Colleges and Universities (HBCUs) remain limited. This study examined the relationships among T2D awareness, healthy eating patterns, and anthropometric risk factors in African American college students attending an HBCU.

**Methods:**

A cross-sectional study was conducted among 211 African American students (146 women, 65 men) aged 18–40 years at Morgan State University, Maryland. T2D awareness was assessed using the 24-item Diabetes Knowledge Questionnaire (DKQ-24), nutrition literacy using the Short Nutrition Literacy Scale (S-NutLit), and dietary intake using the Block Food Frequency Questionnaire, from which a healthy eating pattern score was derived. Anthropometric measurements, including height, weight, body mass index (BMI), waist and hip circumference, and body composition by bioelectrical impedance analysis, were obtained using standardized procedures. Pearson product-moment correlations were performed using SPSS version 25.0, with statistical significance defined as *p* < 0.05.

**Results:**

Mean BMI was 26.8 ± 6.8 kg/m^2^, and 26.5% of participants were classified as obese. Elevated body fat was observed in 45.9% of women and 61.5% of men. Greater T2D awareness was modestly and inversely associated with BMI (*r* = −0.141, *p* = 0.041). Age was positively associated with adherence to the healthy eating pattern (*r* = 0.334, *p* = 0.001), whereas year in college was not associated with T2D awareness or dietary behavior. Among the small subgroup of men with elevated waist circumference (> 102 cm), healthier eating was strongly associated with lower waist circumference (*r* = −0.850, *p* = 0.008).

**Conclusions:**

A substantial proportion of African American students attending this HBCU exhibited anthropometric markers associated with increased T2D risk. Greater diabetes awareness and healthier dietary patterns were associated with more favorable anthropometric profiles, although the observed associations were generally modest. These findings support the need for culturally relevant nutrition education and lifestyle interventions within HBCU settings to promote diabetes prevention and improve long-term cardiometabolic health.

## 1. Introduction

Diabetes mellitus is a major public health challenge and remains one of the leading causes of morbidity and mortality in the United States, contributing to more than 100,000 deaths annually [1, 2]. The disease is associated with serious long-term complications, including cardiovascular disease, chronic kidney disease, peripheral neuropathy, retinopathy, and lower-extremity amputations, which substantially reduce quality of life and impose a considerable economic burden on the healthcare system [2, 3]. The estimated annual cost of diabetes in the United States exceeds $327 billion in direct and indirect expenditures, underscoring the urgent need for effective prevention strategies that target modifiable risk factors and populations at elevated risk [4]. As the prevalence of diabetes continues to increase, identifying vulnerable populations and opportunities for early prevention has become an important public health priority [5, 6].

Type 2 diabetes (T2D) accounts for approximately 90–95% of all diagnosed diabetes cases and was once considered a disease primarily affecting middle-aged and older adults [1, 7]. However, over the past two decades, its incidence has increased markedly among adolescents and young adults [8, 9]. This trend is particularly concerning because early-onset T2D is associated with a longer disease duration, more rapid progression, and a greater lifetime risk of both microvascular and macrovascular complications than later-onset disease [8, 10]. These observations highlight the importance of identifying modifiable risk factors and implementing preventive strategies during early adulthood, before irreversible metabolic complications develop.

Substantial racial and ethnic disparities persist in the prevalence, management, and outcomes of T2D in the United States. African American adults are approximately 60% more likely to be diagnosed with diabetes than non-Hispanic White adults and nearly twice as likely to die from diabetes-related complications [11, 12]. They also experience disproportionately higher rates of diabetes-related end-stage renal disease, lower-extremity amputation, and cardiovascular mortality [12, 13]. These disparities arise from a complex interplay of socioeconomic, behavioral, environmental, cultural, and biological factors, including limited access to healthcare, food insecurity, lower-quality dietary patterns, and chronic psychosocial stress [3, 14]. Importantly, landmark prevention trials such as the Diabetes Prevention Program (DPP) have demonstrated that intensive lifestyle interventions targeting diet and physical activity can reduce T2D incidence by up to 58% among high-risk adults, with benefits sustained for more than a decade [15]. Collectively, these findings underscore the need for targeted prevention strategies that address modifiable risk factors early in life, particularly among populations disproportionately affected by T2D [3, 10].Diet and lifestyle behaviors are among the most important modifiable risk factors for T2D [16, 17]. Dietary patterns characterized by high intakes of refined carbohydrates, added sugars, processed meats, and saturated fats, together with low consumption of fruits, vegetables, and whole grains, are consistently associated with insulin resistance, obesity, and increased T2D risk [18, 19]. In contrast, healthy dietary patterns, including the Mediterranean and Dietary Approaches to Stop Hypertension (DASH) diets, have been associated with a substantially lower risk of T2D [20, 21]. Emerging evidence also suggests that specific dietary components, such as dietary fiber, whole grains, and unsaturated fatty acids, contribute to improved glycemic control and insulin sensitivity [22, 23]. In addition to dietary factors, sugar-sweetened beverage consumption, sedentary behavior, and weight gain further increase T2D risk through their adverse effects on adiposity and glucose metabolism [19, 24].

College students represent a population at increased risk for developing unhealthy lifestyle behaviors during the transition to independent adulthood. This period is often characterized by greater autonomy in food choices, increased reliance on convenience and fast foods, irregular meal patterns, reduced physical activity, increased alcohol consumption, and higher levels of psychological stress [25, 26]. Collectively, these behavioral changes contribute to weight gain, increased adiposity, and the early development of metabolic risk factors that may persist into adulthood [27, 28]. Consistent with these observations, reports from the American College Health Association indicate that a substantial proportion of college students do not meet national recommendations for healthy eating and physical activity, while the prevalence of overweight and obesity continues to increase during the college years [29].

Despite growing interest in T2D risk factors among young adults, African American college students remain underrepresented in the literature. Previous studies conducted in general college populations have shown that many students underestimate their personal risk for T2D despite the presence of established risk factors, including elevated BMI, increased waist circumference, and physical inactivity [30, 31]. However, most of these studies have been conducted in predominantly White or ethnically diverse student populations, limiting their applicability to African American students, who experience a disproportionately higher burden of T2D [32, 33]. Research conducted at Historically Black Colleges and Universities (HBCUs) provides a unique opportunity to examine diabetes awareness, dietary behaviors, and anthropometric risk factors within a population at elevated risk while considering the cultural and environmental context that may influence health behaviors [32, 34]. A better understanding of these relationships is essential for developing culturally relevant, evidence-based interventions that target modifiable risk factors during early adulthood and help reduce future T2D risk in this underserved population [34, 35].

Therefore, this study aimed to examine the relationships among dietary patterns, T2D awareness, nutrition literacy, and anthropometric risk factors among African American students attending Morgan State University, a Historically Black College and University (HBCU) in Baltimore, Maryland. By integrating validated measures of diabetes awareness, nutrition literacy, dietary intake, and objectively measured anthropometric indicators, this study provides a comprehensive assessment of modifiable factors associated with T2D risk in an understudied population. We hypothesized that (1) less healthy dietary patterns would be associated with higher body mass index (BMI) and waist circumference; (2) greater awareness of T2D would be associated with more favorable anthropometric profiles; and (3) year in college would not be significantly associated with T2D awareness or healthy eating patterns, suggesting that age-related maturation rather than progression through the college environment may be more strongly associated with health behaviors during early adulthood.

## 2. Materials and Methods

### 2.1. Study Design and Participants

This cross-sectional study was conducted at Morgan State University, a Historically Black College and University (HBCU) in Baltimore, Maryland. A total of 211 African American students (146 women and 65 men) aged 18–40 years were enrolled. Eligible participants were self-identified African American men and women within the specified age range, and no BMI eligibility criteria were applied. Individuals who were pregnant or reported a previous diagnosis of diabetes mellitus were excluded from participation.

Participants were recruited through multiple campus-based approaches, including printed flyers, classroom announcements, university-wide email communications, and postings on the university's Canvas learning management system. The study protocol was approved by the Institutional Review Board of Morgan State University, and written informed consent was obtained from all participants before data collection.

### 2.2. Data Collection

Data were collected using structured questionnaires comprising previously validated instruments to assess diabetes awareness, nutrition literacy, and dietary behaviors related to T2D risk. All questionnaires were self-administered in a quiet on-campus setting and required approximately 30–40 minutes to complete.

#### Awareness of Type 2 Diabetes.

Awareness of T2D was assessed using the Diabetes Knowledge Questionnaire (DKQ-24), a validated 24-item instrument originally developed for the Starr County Diabetes Education Study. The DKQ-24 demonstrated a reliability coefficient of 0.78 and established construct validity in both English- and Spanish-speaking populations [36]. The questionnaire assesses knowledge of T2D risk factors, symptoms, complications, and prevention strategies.

#### Nutrition Literacy.

Nutrition literacy was assessed using the Short Nutrition Literacy Scale (S-NutLit), an 11-item self-administered instrument developed and validated by Guttersrud and colleagues [37]. The S-NutLit evaluates two complementary domains of nutrition literacy: information skills (8 items; Cronbach's α = 0.83) and expert skills (3 items; Cronbach's α = 0.79). Responses are recorded on a 5-point Likert scale ranging from 1 ("totally disagree") to 5 ("totally agree"), yielding total scores from 11 to 55, with higher scores indicating greater nutrition literacy.

#### Food Frequency Questionnaire.

Dietary intake was assessed using an adapted version of the Block Food Frequency Questionnaire (FFQ), a validated instrument widely used in nutritional epidemiology and developed using dietary recall data from the National Health and Nutrition Examination Survey (NHANES) [38, 39]. Participants reported the frequency of consumption of foods representing major dietary sources of energy and nutrients relevant to T2D risk. A healthy eating pattern score was derived from the FFQ to reflect greater consumption of fruits, vegetables, whole grains, and dietary fiber, together with lower consumption of refined carbohydrates, added sugars, and sugar-sweetened beverages [40].

### 2.3. Anthropometric Measurements

Anthropometric measurements were obtained by trained research staff using standardized procedures consistent with the National Health and Nutrition Examination Survey (NHANES) Anthropometry Procedures Manual [40]. Height and body weight were measured using a SECA 763 digital stadiometer/scale (SECA GmbH & Co. KG, Hamburg, Germany), with participants wearing light clothing and no shoes. BMI was calculated as weight in kilograms divided by height in meters squared (kg/m^2^) and classified according to World Health Organization (WHO) criteria as underweight (< 18.5 kg/m^2^), normal weight (18.5–24.9 kg/m^2^), overweight (25.0–29.9 kg/m^2^), or obese (≥ 30.0 kg/m^2^) [41].

Body composition, including percentage body fat and fat-free mass, was assessed using a Tanita SC-240 IM Ironman body composition analyzer (Tanita Corporation, Tokyo, Japan), which uses bioelectrical impedance analysis and has been validated against reference methods in young adult populations [42]. Waist and hip circumferences were measured to the nearest 0.1 cm using a non-stretchable measuring tape. Waist circumference was measured at the midpoint between the lowest rib and the iliac crest, whereas hip circumference was measured at the widest portion of the buttocks, in accordance with WHO recommendations [43]. Elevated waist circumference was defined as > 102 cm (40 inches) for men and > 88 cm (35 inches) for women, consistent with established WHO criteria for increased cardiometabolic risk [43]. All anthropometric measurements were obtained in duplicate, and the mean of the two measurements was used for statistical analyses.

### 2.4. Statistical Analysis

All statistical analyses were performed using IBM SPSS Statistics version 25.0 (IBM Corp., Armonk, NY, USA). Descriptive statistics, including means, standard deviations, frequencies, and percentages, were used to summarize participant characteristics. The distribution of continuous variables was evaluated by visual inspection of histograms and quantile-quantile (Q-Q) plots to assess the assumption of normality. Pearson product-moment correlation coefficients were calculated to examine the relationships among T2D awareness (DKQ-24), nutrition literacy (S-NutLit), the healthy eating pattern score derived from the FFQ, age, year in college, and anthropometric risk factors, including BMI, waist circumference, hip circumference, waist-to-hip ratio, and body fat percentage. Correlation coefficients were interpreted according to Cohen's guidelines as small (0.10), medium (0.30), and large (0.50) effect sizes [44]. Sex-stratified subgroup analyses were performed among participants with elevated body fat (> 30% in women and > 20% in men) and elevated waist circumference (> 88 cm in women and > 102 cm in men). All statistical tests were two-tailed, and a *p* value < 0.05 was considered statistically significant.

## 3. Results

### 3.1. Participant Characteristics

A total of 211 African American students participated in the study, including 146 women (69.2%) and 65 men (30.8%). The mean age of participants was 20.3 ± 3.1 years, and 99.1% were single. Participant sociodemographic and anthropometric characteristics are presented in **Table 1**.

The mean BMI was 26.8 ± 6.8 kg/m^2^, placing the average participant in the overweight category, and 56 participants (26.5%) were classified as obese (BMI ≥ 30.0 kg/m^2^). Mean waist circumference was 86.9 ± 15.2 cm (34.2 ± 6.0 inches), and mean hip circumference was 99.8 ± 15.0 cm (39.3 ± 5.9 inches). Elevated waist circumference (> 88 cm in women and > 102 cm in men) was observed in 46 women (31.5%) and 8 men (12.3%). Mean body fat percentage was 27.6 ± 12.7%, with elevated body fat observed in 45.9% of women and 61.5% of men.

### 3.2. Type 2 Diabetes Awareness and Anthropometric Risk Factors

Pearson correlation coefficients between T2D awareness (DKQ-24) and anthropometric risk factors are presented in **Table 2**. T2D awareness was modestly and inversely correlated with BMI (*r* = −0.141, *p* = 0.041), indicating that participants with greater diabetes awareness tended to have lower BMI. Inverse correlations were also observed between T2D awareness and waist circumference (*r* = −0.117, *p* = 0.091), waist-to-hip ratio (*r* = −0.112, *p* = 0.104), hip circumference (*r* = −0.039, *p* = 0.569), body fat percentage (*r* = −0.039, *p* = 0.569), and obesity status (*r* = −0.061, *p* = 0.829); however, these associations were not statistically significant.

Sex-stratified subgroup analyses showed no statistically significant associations between T2D awareness and body fat percentage among women with elevated body fat (*r* = −0.021, *p* = 0.863) or men with elevated body fat (*r* = −0.082, *p* = 0.614). Similarly, no significant associations were observed among women (*r* = −0.220, *p* = 0.143) or men (*r* = −0.104, *p* = 0.807) with elevated waist circumference.

### 3.3. Healthy Eating Pattern and Anthropometric Risk Factors

Correlations between the healthy eating pattern score and anthropometric risk factors are presented in **Table 3**. Inverse correlations were observed between the healthy eating pattern score and BMI (*r* = −0.125, *p* = 0.073), waist-to-hip ratio (*r* = −0.123, *p* = 0.078), waist circumference (*r* = −0.105, *p* = 0.133), hip circumference (*r* = −0.034, *p* = 0.625), body fat percentage (*r* = −0.048, *p* = 0.496), and obesity status (*r* = −0.118, *p* = 0.385); however, none of these associations reached statistical significance in the overall study population.

Among participants with elevated body fat or waist circumference, inverse but non-significant associations were observed among women with elevated body fat (*r* = −0.118, *p* = 0.340), women with elevated waist circumference (*r* = −0.007, *p* = 0.966), and men with elevated body fat (*r* = −0.218, *p* = 0.183). In contrast, among the subgroup of men with elevated waist circumference (> 102 cm), the healthy eating pattern score was strongly and inversely correlated with waist circumference (*r* = −0.850, *p* = 0.008).

### 3.4. Age, Year in College, and Health Behaviors

Correlations among age, year in college, T2D awareness, and the healthy eating pattern score are presented in **Table 4**. Age was moderately and positively correlated with the healthy eating pattern score (*r* = 0.334, *p* = 0.001), indicating that older participants tended to report healthier dietary patterns. In contrast, year in college was not significantly associated with T2D awareness (*r* = 0.001, *p* = 0.996) or the healthy eating pattern score (*r* = 0.129, *p* = 0.063). Similarly, age was not significantly associated with T2D awareness (*r* = −0.055, *p* = 0.425).

## 4. Discussion

This cross-sectional study examined the relationships among dietary patterns, T2D awareness, and anthropometric risk factors among 211 African American students attending an HBCU in Maryland. The principal findings were that: (i) a substantial proportion of participants exhibited anthropometric indicators associated with elevated T2D risk, with a mean BMI in the overweight range (26.8 ± 6.8 kg/m^2^), 26.5% classified as obese, and elevated body fat observed in 45.9% of women and 61.5% of men [1, 2]; (ii) greater T2D awareness was modestly and inversely associated with BMI; (iii) healthier eating patterns were associated with smaller waist circumference among the subgroup of men with elevated central adiposity; and (iv) age, but not year in college, was positively associated with healthier dietary patterns. Collectively, these findings extend the limited evidence describing T2D-related risk factors among African American students attending HBCUs and highlight the potential value of early, culturally relevant nutrition and lifestyle interventions in this population [27, 30, 31, 32, 33].

### 4.1. Comparison with Prior College-Based Studies

The anthropometric findings of the present study are generally consistent with those reported in previous studies of U.S. college students. Antwi and colleagues, in a pilot study of 44 students at an upstate New York college, reported that 36.4% had elevated BMI, 56.8% had low HDL cholesterol, and 45.5% had impaired fasting glucose, with physical inactivity (61.4%) identified as the most common modifiable risk factor [31]. Although biochemical markers were not assessed in the present study, the prevalence of obesity (26.5%) and elevated body fat (45.9% of women and 61.5% of men) indicates that anthropometric risk factors associated with T2D are common among African American students attending this HBCU [26, 30]. These findings are particularly relevant given that African Americans experience a substantially higher lifetime risk of T2D than non-Hispanic White adults and often develop the disease at lower BMI thresholds [2, 3, 11].

The modest inverse association observed between T2D awareness and BMI (*r* = − 0.141) is consistent with previous studies reporting that greater diabetes knowledge is associated with more favorable anthropometric outcomes among young adults [25, 30, 31]. However, the relatively small effect size suggests that diabetes awareness alone is unlikely to explain differences in body weight. Behavioral change is influenced by multiple factors beyond knowledge, including motivation, self-efficacy, access to healthy foods, and the surrounding food environment [25, 31]. In addition, the high prevalence of diabetes within many African American communities may reduce variability in diabetes awareness, thereby attenuating observed associations [32, 34]. Nevertheless, the consistent inverse direction of the associations observed across several anthropometric measures is in agreement with previous evidence suggesting that nutrition education and diabetes literacy may contribute to healthier weight-related outcomes when combined with broader lifestyle interventions [27, 30].

### 4.2. Healthy Eating and Central Adiposity in Men

A second important finding was the strong inverse association between the healthy eating pattern score and waist circumference among the small subgroup of men with elevated central adiposity (> 102 cm; *r* = −0.850, *p* = 0.008). This observation is consistent with previous evidence demonstrating that dietary patterns characterized by higher intakes of whole grains, dietary fiber, fruits, and vegetables, together with lower consumption of refined carbohydrates, are associated with reduced central adiposity and lower T2D risk [17, 18, 22]. Large prospective cohort studies and meta-analyses have similarly reported that adherence to healthy dietary patterns is associated with smaller waist circumference and a reduced risk of metabolic disease [18, 20, 22]. Findings from the Diabetes Prevention Program further demonstrate that dietary improvement, combined with increased physical activity, can substantially reduce the incidence of T2D among adults at elevated metabolic risk [15].

Although the observed association was strong, it should be interpreted with caution because it was based on a very small subgroup of participants (*n* = 8). Correlation estimates derived from small samples are inherently less stable and are associated with wider confidence intervals [44]. Therefore, this finding should be considered exploratory and hypothesis-generating rather than confirmatory, and replication in larger cohorts of African American men attending HBCUs is warranted.

### 4.3. Age, Maturation, and the College Environment

The positive association between age and the healthy eating pattern score (*r* = 0.334, *p* = 0.001), together with the absence of a significant association between year in college and either T2D awareness or dietary behavior, suggests that maturation rather than progression through the college environment may play a greater role in shaping dietary behaviors during early adulthood. Similar findings have been reported by Sogari and colleagues and Deliens and colleagues, who attributed improvements in dietary behaviors among young adults to increasing autonomy, greater exposure to nutrition information, and ongoing identity development rather than to formal academic coursework [25, 26]. These observations suggest that healthy dietary behaviors may evolve with age and life experience rather than as a direct consequence of advancing through the college curriculum.

Although colleges and universities provide opportunities to promote healthy lifestyles, our findings indicate that progression through college alone may be insufficient to improve diabetes awareness or dietary behaviors. These findings highlight the potential value of implementing targeted nutrition education and health promotion programs that actively support healthy lifestyle behaviors throughout the college years, particularly in settings serving populations at elevated risk for T2D.

### 4.4. Implications for Prevention in HBCU Settings

The high prevalence of overweight, obesity, elevated body fat, and central adiposity observed in this study underscores the need for early T2D prevention strategies among African American college students attending HBCUs [32, 33, 34, 35]. Although the observed associations between diabetes awareness, healthy eating patterns, and anthropometric measures were generally modest, they suggest that improving nutrition knowledge and promoting healthier dietary behaviors may contribute to reducing modifiable risk factors for T2D. HBCUs provide a unique and culturally relevant setting for implementing nutrition education and lifestyle interventions that can be integrated into existing student wellness initiatives [32, 33, 34, 35].

Evidence from the Diabetes Prevention Program has demonstrated that lifestyle interventions incorporating dietary modification, increased physical activity, and modest weight loss substantially reduce the incidence of T2D among high-risk adults [15]. While younger populations remain underrepresented in this evidence base, the college years represent an important opportunity to establish lifelong healthy behaviors before the onset of chronic disease. Consequently, culturally tailored nutrition education and health promotion programs delivered through HBCU wellness infrastructure may represent a promising strategy to reduce future T2D risk and promote health equity among African American college students [17, 22].

### 4.5. Strengths and Limitations

This study has several important strengths. To our knowledge, it is among the few studies to simultaneously evaluate diabetes awareness, nutrition literacy, dietary patterns, and objectively measured anthropometric and body composition indicators among African American students attending a Historically Black College and University. The use of validated instruments, including the DKQ-24 for diabetes awareness, the S-NutLit for nutrition literacy, and the Block Food Frequency Questionnaire for dietary assessment, enhances the reliability and comparability of the findings. Anthropometric measurements were obtained using standardized NHANES procedures, and body composition was assessed by bioelectrical impedance analysis rather than relying on self-reported height and weight, thereby reducing measurement error commonly encountered in college-based research. In addition, sex-stratified analyses enabled the identification of subgroup-specific patterns that may not have been evident in the overall study population.

Several limitations should also be acknowledged. First, the cross-sectional design precludes causal inference, and the observed associations reflect relationships rather than cause-and-effect. Second, the modest effect sizes suggest that diabetes awareness and self-reported dietary behaviors represent only some of the factors associated with anthropometric outcomes. Other important determinants, including socioeconomic status, the food environment, physical activity, sleep, psychosocial stress, and genetic predisposition, were not evaluated and may have influenced the observed relationships. Third, dietary intake and behavioral measures were self-reported and therefore subject to recall and social desirability bias. Fourth, although the S-NutLit has been widely used, its psychometric performance in young adult populations warrants further investigation [37]. Finally, the subgroup of men with elevated waist circumference was small (*n* = 8); therefore, the observed association between the healthy eating pattern score and waist circumference should be interpreted as exploratory and requires confirmation in larger studies [44]. Because this study was conducted at a single HBCU in Maryland, the findings may not be generalizable to African American students attending other institutions or to young adults in non-college settings.

## 5. Conclusion

Among African American students attending an HBCU, anthropometric markers associated with increased T2D risk were common, highlighting the importance of early prevention in this population. Greater diabetes awareness and healthier dietary patterns were associated with more favorable anthropometric profiles, although these associations were generally modest and should be interpreted within the context of the cross-sectional study design. These findings support the integration of nutrition education and behaviorally grounded lifestyle interventions into HBCU wellness programs to address modifiable risk factors during early adulthood. Leveraging the trusted and culturally affirming environment of HBCUs may provide an important opportunity to reduce future T2D risk and advance health equity in a population disproportionately affected by diabetes [2, 3, 32, 33, 34, 35].

## Supplementary Material

This is a list of supplementary files associated with this preprint. Click to download.

- floatimage1.jpeg
- floatimage2.jpeg
- floatimage3.jpeg
- floatimage4.jpeg

Tables 1 to 4 are available in the Supplementary Files section.
